# The shape of the glucose response curve during an oral glucose tolerance test heralds β–cell function in a large Chinese population

**DOI:** 10.1186/s12902-019-0446-4

**Published:** 2019-11-05

**Authors:** Xinqi Cheng, Na Yang, Yuxiu Li, Qi Sun, Ling Qiu, Lingling Xu, Fan Ping, Wei Li, Huabing Zhang

**Affiliations:** 1 Department of Clinical Laboratory, Peking Union Medical College Hospital, Chinese Academy of Medical Sciences & Peking Union Medical College, Beijing, China; 2 Department of Endocrinology, Key Laboratory of Endocrinology, Ministry of Health, Peking Union Medical College Hospital, Chinese Academy of Medical Sciences & Peking Union Medical College, Beijing, China

**Keywords:** OGTT, Glucose response curve, Chinese

## Abstract

**Background:**

The shape of the glucose response curve during an oral glucose tolerance test (OGTT) can predict β-cell function and insulin resistance. However, there have been few studies conducted on Chinese people. Thus, we aimed to verify the usefulness of the glucose response curve in a large Chinese population.

**Methods:**

A total of 9059 OGTT (3-h tests) were categorized into either a monophasic or a multiphasic group based on the shape of the glucose response. Homeostasis model assessments of fasting insulin resistance, the Matsuda Index, the insulinogenic index, and the disposition index were assessed by plasma glucose and serum insulin concentration obtained at fasting or during an OGTT.

**Results:**

The shape of the OGTT glucose response curve was monophasic in 87.3% and multiphasic in 12.7% of participants. Individuals in the multiphasic group were younger compared to those in the monophasic group (38.6 ± 13.6 vs. 35.4 ± 13.5, *P* < 0.001). Individuals in the monophasic group had significantly higher fasting plasma glucose (FPG 5.6 ± 13.5 vs. 5.2 ± 0.6, *P* < 0.001), fasting insulin (FINS 14.8 ± 8.7 vs. 13.5 ± 7.9, *P* < 0.01), and homeostasis model assessment of insulin resistance (HOMA-IR 3.8 ± 2.6 vs. 3.1 ± 2.0, *P* < 0.001) and impaired β-cell function (disposition index 12.7 ± 14.1 vs. 16.6 ± 17.8, *P* < 0.001) compared to those in the multiphasic group.

**Conclusion:**

The monophasic OGTT glucose response curve could reflect impaired β-cell function in a large Chinese population.

## Background

Diabetes mellitus (DM) has been categorized as a complex and multifactorial metabolic condition [1]. Many factors play important roles in the development of glucose intolerance in individuals with type 2 diabetes (T2D), such as impaired insulin secretion and insulin resistance [2, 3]. It is widely accepted that the gold standard method for evaluating insulin action is hyperinsulinemic-euglycemic clamp [4]. Because this method is invasive, complicated, and expensive, its application in clinical practice is limited. The oral glucose tolerance test (OGTT) has been used to diagnose diabetes or to capture the impaired fasting glucose (IFG) and impaired glucose tolerance (IGT) based on the fasting plasma glucose (FPG) and 2-h plasma glucose (2hPG) [5]. Although β-cell function and insulin action can be obtained through calculating a series of formulas, such as the insulinogenic index or Matsuda Index during the OGTT, they are not intuitive. The OGTT glucose response curve could be a novel and intuitive biomarker to identify early metabolic risk [6]. Recent cross-sectional studies [6–13] showed that OGTT response curves, either monophasic or biphasic, can not only indicate β-cell function and insulin resistance but also differentiate diabetes risk. Those studies revealed that individuals with a monophasic curve tended to have worse insulin sensitivity and β-cell function. A recent prospective study demonstrated that individuals with a biphasic curve developed T2D at a lower rate than those with a monophasic curve, independent of FPG and/or 2hPG [14].

However, the scale of these studies was generally small, with a maximum of hundreds of subjects included, and only one study was conducted in an Asian population [11]. These studies mainly focused on people without diabetes. No studies showed a relationship between age and the shape of the glucose response curve. In addition, few studies reported the dynamic change of glucose response curves and their relationship with baseline β-cell function and insulin sensitivity.

Therefore, the purposes of this study were as follows: 1) to verify the utility of the OGTT glucose response curve in predicting β-cell function and insulin sensitivity in a large Chinese population with varying statuses of glucose tolerance; 2) to examine the relationship between age and the shape of glucose response curves; and 3) to assess if the shape of glucose response curves changed dynamically over time and whether the change was related to baseline β-cell function and insulin sensitivity.

## Methods

### Subjects

We retrospectively analyzed data of individuals who were tested with a 3-h OGTT and with complete glucose and insulin testing in Peking Union Medical College Hospital from August 2011 to January 2018. Participants were excluded from the study if any of the following criteria were met: a) missing demographic information (age or sex); b) under 18 years old and over 18 years old who did not receive 75 g glucose for OGTT; c) FPG < 3.9 mmol/L; d) history of diabetes; e) FINS > 60 μIU/ml or serum insulin level > 300 μIU/ml at any point of OGTT, because the upper limit was 300 μIU/ml; e) 30-min plasma glucose ≤0-min plasma glucose or 30-min serum insulin ≤0-min serum insulin, to ensure insulinogenic index could be calculated; and g) shapes of the curve could not be classified.

### Blood sampling and OGTT

After an overnight fast, participants underwent a 3-h OGTT with the ingestion of 75 g glucose. Venous blood samples were obtained at 0, 30, 60, 120, and 180 min. Plasma glucose was measured by the hexokinase method using a Beckman AU2700 analyzer (Beckman Coulter, Brea, CA, USA). Serum insulin was assessed by chemiluminescence immunoassay using a Siemens ADIVA Centaur XP analyzer (Siemens Healthcare Diagnostics Inc., Tarrytown, NY, USA). The glucose and insulin assays were standardized to NIST SRM 965 and WHO 1st IRP 66/304, respectively. The repeatability and within laboratory coefficient variations were < 5%.

### Classification of glucose tolerance status

According to the World Health Organization definition [5, 15, 16], normal glucose tolerance (NGT) was defined as FPG < 6.1 mmol/L and 2-h plasma glucose < 7.8 mmol/L. Prediabetes was defined as having IFG (FPG: 6.1–6.9 mmol/L) and/or IGT (2-h plasma glucose: 7.8–11.0 mmol/L). Diabetes was defined as having FPG ≥7.0 mmol/l and/or 2-h plasma glucose ≥11.1 mmol/L.

### Classification of glucose curve shapes

The shapes were classified in line with previous studies [17]. A monophasic response curve was determined by a gradual increase in glucose concentrations until a peak was reached, followed by a subsequent decrease in glucose of ≥0.25 mmol/L. A biphasic response curve was defined by the second rise in glucose concentrations of ≥0.25 mmol/L. A triphasic response curve was defined by two complete peaks of the plasma glucose curve, with every rise and decrease in glucose concentrations of ≥0.25 mmol/L. The latter two were collectively called multiphasic response curve. This was done with a plasma glucose threshold of 0.25 mmol/L to minimize fluctuations in glucose concentrations, which may be caused by the method of glucose analysis rather than by physiological reasons.

### Calculation of variables

Areas under the glucose and insulin curves were calculated with the trapezoidal rule [10]. Insulin action was estimated by the homeostasis model assessment for insulin resistance (HOMA-IR) and the whole-body insulin sensitivity index of Matsuda. HOMA-IR = (I_0_-G_0_)/22.5, with glucose and insulin expressed as mmol/L and mUI/ml, respectively [18]. The Matsuda Index = 10,000/√[(fasting glucose (mg/dl) × FINS (μU/ml)) × (mean glucose (mg/dl) × mean insulin (μU/ml)] [19]. Insulin secretion was estimated by the insulinogenic index. The insulinogenic index was calculated using fasting and 30-min insulin and glucose concentrations [20]. β-cell function was estimated by the disposition index as the product of insulinogenic index and HOMA-IR [21].

### Statistical analysis

Summary statistics were calculated using frequencies and proportions for categorical data and means (standard deviations) for continuous variables. Kruskal-Wallis, Pearson χ^2^, and unpaired Student’s t-tests were used for comparisons. Analysis of covariance was used to compare two glucose response curve groups (monophasic vs. multiphasic) after adjusting for the potential confounding effects (age, sex, glycemic status). A two-sided *p*-value < 0.05 was considered to indicate statistical significance.

All data were analyzed using IBM SPSS Statistics, version 25. The authors have full access to and take full responsibility for the integrity of the data. The manuscript has been read and approved by all the authors.

## Results

### Baseline characteristics according to glucose curve shapes

**Fig. 1:**
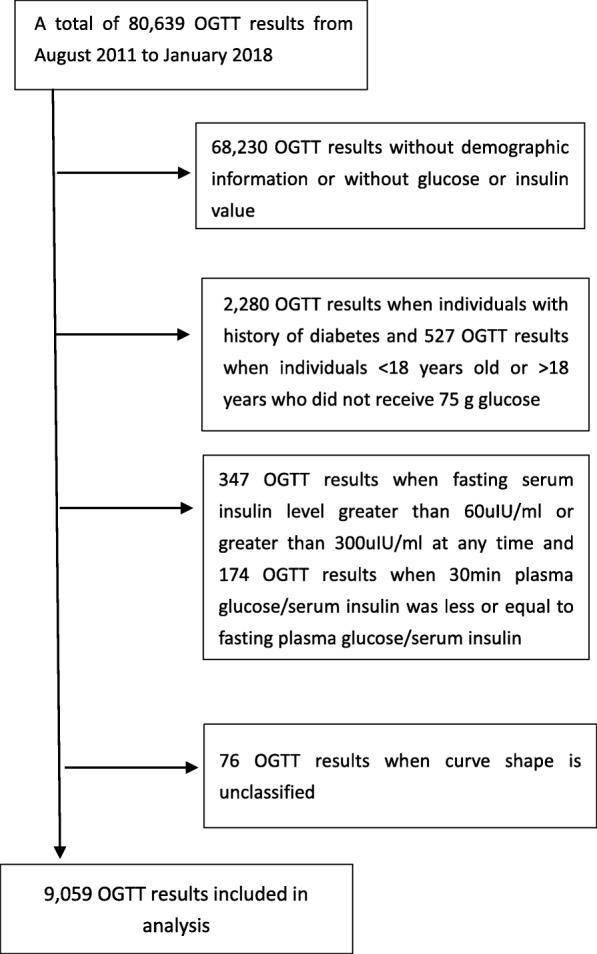
Study Flow Diagram OGTT: oral glucose tolerance test

**Fig. 2:**
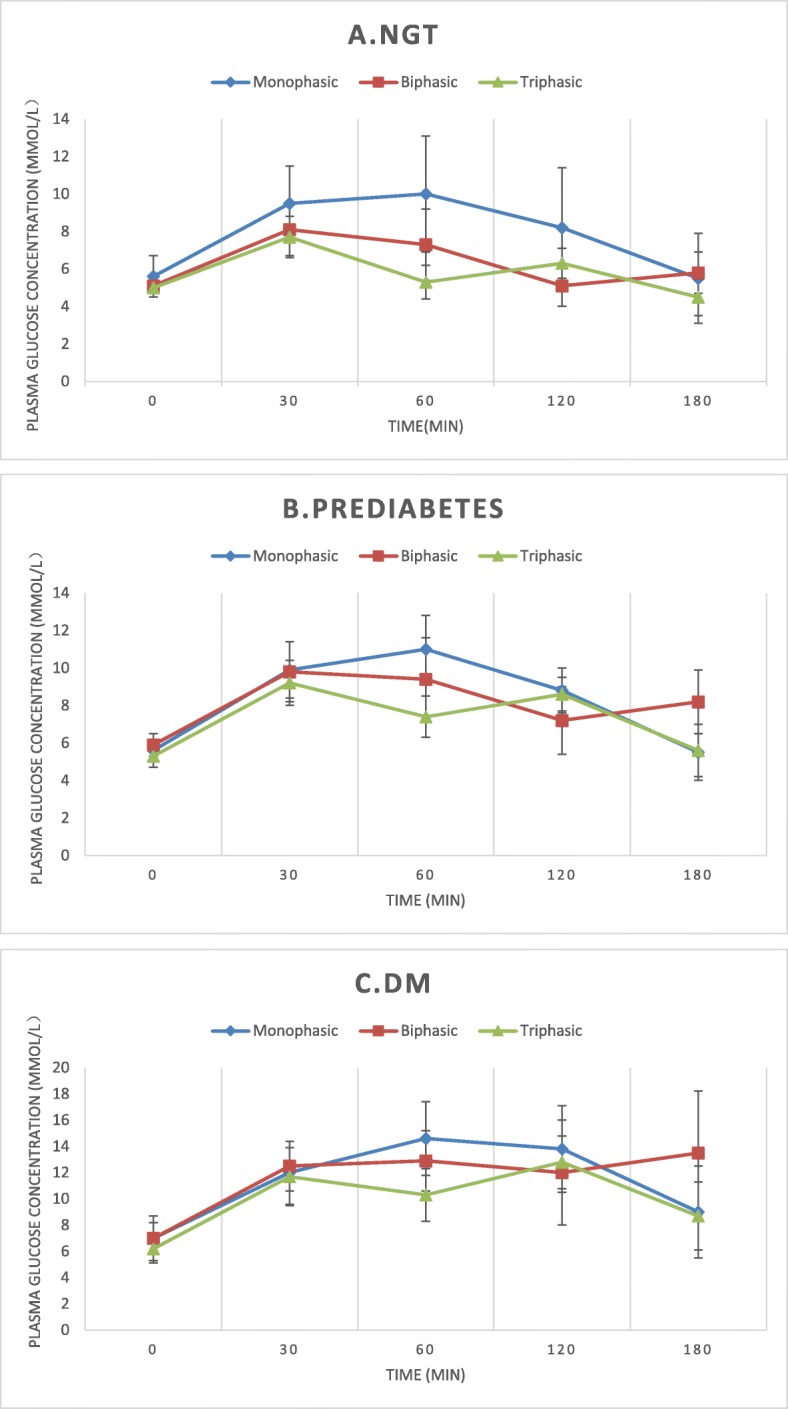
Glucose during a 3-h OGTT in monophasic, biphasic and triphasic groups among different glycemic status. NGT: normal glucose tolerance, DM: diabetes mellitus

A total of 9059 OGTTs in 8391 study individuals were included in the final analysis (Fig. 1). The baseline age was 38.2 ± 13.6 years, and 74.4% were female. In terms of the shape of the participants’ OGTT glucose response curve, 87.3% were monophasic, 6.1% were biphasic, and 6.6% were triphasic. Although the individuals with a triphasic curve had better insulin sensitivity and β-cell function than those with a biphasic curve (Table 1), in view of the limited cases, the triphasic group and the biphasic group were collectively referred to as the multiphasic group. Physical and glucose metabolic characteristics of participants with monophasic and multiphasic curves are presented in Table 1. Figure 2 illustrates the average glucose at each point in the monophasic, biphasic, and triphasic curves. The monophasic group exhibited significantly higher FPG (5.6 ± 13.5 vs. 5.2 ± 0.6), 2hPG (8.2 ± 3.2 vs. 6.3 ± 2.0), fasting serum insulin (FINS), and 2 h serum insulin (2 h INS) than the multiphasic group. The monophasic group had a significantly higher HOMA-IR (3.8 ± 2.6 vs. 3.1 ± 2.0) and lower Matsuda Index (2.9 ± 1.9 vs. 3.8 ± 2.5) and insulinogenic index (25.1 ± 23.0 vs. 16.6 ± 17.8). Disposition index, the indicator of β-cell function, was nearly 42% lower in the monophasic group (Table 1). These differences remained significant after adjusting for sex and age.

**Table 1:** Demographic and metabolic characteristics of 9059 participants with monophasic versus multiphasic OGTT glucose response curve and 1150 participants with biphasic versus triphasic

Variables	Monophasic group (*n* = 7909)	Multiphasic group (*n* = 1150)	*P* value	Biphasic group (*n* = 548)	Triphasic group (*n* = 602)	*P* value
Age (years)	38.6 ± 13.6	35.4 ± 13.5	< 0.001	35.1 ± 13.7	35.8 ± 13.2	0.391
Sex (male/female), n%	2100 (26.6)/5809 (73.4)	219 (19.0)/931 (81.0)	< 0.001	106 (19.3)/442 (80.7)	113 (18.8)/489 (81.2)	0.805
FBG (mmol/L)	5.6 ± 13.5	5.2 ± 0.6	< 0.001	5.2 ± 0.6	5.2 ± 0.6	0.189
30 min GLU (mmol/L)	9.5 ± 2.0	8.3 ± 1.6	< 0.001	8.4 ± 1.6	8.2 ± 1.6	<0.001
2 h GLU (mmol/L)	8.2 ± 3.2	6.3 ± 2.0	< 0.001	5.4 ± 1.8	7.2 ± 1.9	<0.001
FINS	14.8 ± 8.7	13.5 ± 7.9	< 0.001	13.2 ± 8.4	13.7 ± 7.5	0.328
30 min INS	97.7 ± 59.6	123.5 ± 67.1	< 0.001	105.6 ± 62.0	139.7 ± 67.5	<0.001
2 h INS	106.9 ± 66.9	76.8 ± 57.8	< 0.001	54.7 ± 43.2	96.8 ± 62.0	<0.001
Glycemic status(%)						
NGT	4253 (53.8)	903 (78.5)		486 (88.7)	417 (69.3)	
IFG/IGT/IFG + IGT	2362 (29.9)	206 (17.9)	< 0.001	50 (9.1)	156 (25.9)	<0.001
DM	1294 (16.3)	41 (3.6)		12 (2.2)	29 (4.8)	
Glucose AUC (mg·dL ^−1^· h ^−1^)	1473.5 ± 437.1	1181.2 ± 257.8	< 0.001	1180.0 ± 259.7	1182.4 ± 256.2	0.876
Insulin AUC (mg·dL ^−1^· h ^− 1^)	16,299.7 ± 8297.5	13,968.7 ± 7498.6	< 0.001	13,182.2 ± 7277.7	14,684.7 ± 7629.9	0.001
HOMA-IR	3.8 ± 2.6	3.1 ± 2.0	< 0.001	3.1 ± 2.1	3.2 ± 1.8	0.595
Matsuda Index	2.9 ± 1.9	3.8 ± 2.5	< 0.001	4.0 ± 2.7	3.6 ± 2.3	0.018
Insulinogenic index	25.1 ± 23.0	42.9 ± 37.7	< 0.001	36.0 ± 35.9	49.1 ± 38.2	<0.001
Disposition index	12.7 ± 14.1	16.6 ± 17.8	< 0.001	13.7 ± 15.4	19.2 ± 19.4	<0.001

Values are reported as the mean ± SD or n (%). *FBG* fasting blood glucose, *30 min GLU* glucose at 30 min, *2 h GLU* glucose at 120 min, *FINS* fasting insulin, *30 min INS* insulin at 30 min, *2 h INS* insulin at 1200 min, *NGT* normal glucose tolerance, *IFG* impaired fasting glucose, *IGT* impaired glucose tolerance, *DM* diabetes mellitus, *AUC* area under the curve

### Comparison of the glucose curve shapes among different age groups

**Fig. 3:**
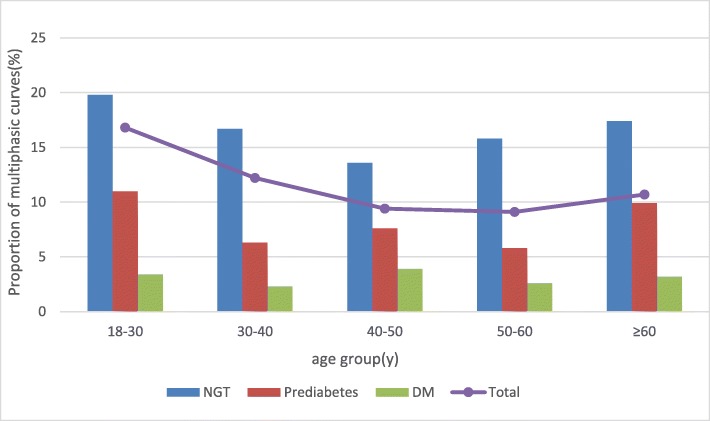
Proportion of multiphasic curves at different age group and glycemic status NGT: normal glucose tolerance, DM: diabetes mellitus

To explore the relationship between age and the shape of the OGTT curve, we divided age into six categories (Fig. 3). We found that a higher proportion of younger people belonged to the multiphasic group, i.e., a significantly higher proportion of participants aged 18 to 30 years (16.8%) were in the multiphasic group compared with all the other, older groups (30–40 years: 12.2%, 40–50 years: 9.4%, 50–60 years: 9.1%, ≥60 years: 10.7%) (*p* < 0.001). Furthermore, we divided each age group into three glycemic stages (NGT, prediabetes, DM) to adjust the effect of age on glycemic status (Fig. 3). When participants were in NGT or prediabetes, younger individuals had a higher proportion of multiphasic curves. However, when the glycemic status progressed to diabetes, quite a low percentage of individuals in all age groups had multiphasic curves.

### Types of glucose curve shape as the indicator for insulin resistance and β-cell function

Surrogate markers of insulin sensitivity and β-cell function including HOMA-IR, Matsuda Index, insulinogenic index, and disposition index were significantly different between the monophasic and multiphasic groups (Table 1). After stratification in glycemic status, the difference in insulinogenic index remained significant at every status of glycemic metabolism. However, the other three indexes no longer showed significant differences in individuals with DM. In addition, no significant differences between the two groups were noted for HOMA-IR in participants with prediabetes (Table 2).

**Table 2:** Demographic and metabolic characteristics of 9059 participants with monophasic versus multiphasic OGTT-glucose response curve in different glycemic status

	NGT(*n* = 5156)			Prediabetes(*n* = 2568)			DM(*n* = 1334)		
	Monophasic (*n* = 4253)	Multiphasic (*n* = 903)	P	Monophasic (*n* = 2362)	Multiphasic (*n* = 206)	P	Monophasic (*n* = 1293)	Multiphasic (*n* = 41)	P
Age (years)	35.1 ± 12.3	33.9 ± 12.5	0.008	41.3 ± 13.6	40.1 ± 15.4	0.213	45.2 ± 13.7	46.3 ± 13.9	0.598
Sex (male/female), n%	963 (22.6)/3290 (77.4)	159 (17.6)/744 (82.4)	0.001	682 (28.9)/1680 (71.1)	53 (25.7)/153 (74.3)	0.338	455 (35.2)/838 (64.8)	7 (17.1)/34 (82.9)	0.016
FBG (mmol/L)	5.1 ± 0.5	5.1 ± 0.4	< 0.001	5.6 ± 0.6	5.5 ± 0.6	< 0.001	7.0 ± 1.7	6.4 ± 1.2	0.943
30 min (mmol/L)	8.5 ± 1.3	7.9 ± 1.3	< 0.001	9.9 ± 1.5	9.3 ± 1.3	< 0.001	11.9 ± 2.4	12.0 ± 2.2	0.962
2hPG (mmol/L)	6.1 ± 0.9	5.6 ± 1.2	< 0.001	7.8 ± 1.7	8.8 ± 1.2	< 0.001	13.8 ± 3.3	12.6 ± 2.7	0.017
FINS	13.8 ± 8.0	13.0 ± 7.7	0.008	15.6 ± 8.5	15.2 ± 8.4	0.578	17.0 ± 10.4	15.3 ± 8.0	0.301
30 min INS	112.0 ± 61.6	124.1 ± 68.0	< 0.001	91.9 ± 54.4	127.9 ± 63.5	< 0.001	61.7 ± 42.8	87.9 ± 53.6	0.003
2 h INS	88.1 ± 57.0	66.7 ± 50.5	< 0.001	133.8 ± 68.7	115.9 ± 67.8	< 0.001	119.9 ± 73.7	100.6 ± 64.3	0.098
Glucose AUC (mg·dL ^−1^· h ^−1^)	1197.1 ± 158.0	1094.1 ± 152.8	< 0.001	1563.7 ± 163.9	1398.0 ± 158.4	< 0.001	2219.0 ± 462.6	2010.2 ± 368.5	0.004
Insulin AUC (mg·dL ^−1^· h ^− 1^)	15,411.0±7733.0	13,198.7±7054.3	< 0.001	18,336.3±8582.0	17,218.6±8506.1	0.073	15,514.8±8888.9	14,599.2±7349.0	0.514
HOMA-IR	3.2 ± 1.9	3.0 ± 1.8	0.002	3.9 ± 2.3	3.7 ± 2.1	0.199	5.4 ± 4.1	4.4 ± 2.8	0.138
Matsuda Index	3.3 ± 1.9	4.1 ± 2.6	< 0.001	2.5 ± 1.6	2.8 ± 1.5	0.004	2.3 ± 1.8	2.6 ± 1.4	0.419
Insulinogenic index	33.1 ± 26.6	46.4 ± 39.6	< 0.001	19.2 ± 13.5	32.9 ± 26.7	< 0.001	9.4 ± 7.8	13.9 ± 10.3	< 0.001
Disposition index	15.1 ± 16.0	17.0 ± 18.2	0.001	11.7 ± 11.7	16.5 ± 17.0	< 0.001	6.9 ± 8.5	7.6 ± 7.1	0.594

Values are reported as the mean ± SD or n (%). *NGT* normal glucose tolerance, *DM* diabetes mellitus, *FBG* fasting blood glucose, *30 min GLU* glucose at 30 min, *2 h GLU* glucose at 120 min, *FINS* fasting insulin, *30 min INS* insulin at 30 min, *2 h INS* insulin at 1200 min, *AUC* area under the curve

### OGTT glucose peak time, nadir time, and insulin peak time in relation to insulin resistant and β-cell function

The multiphasic group could be further divided into two categories by the time when glucose is lowest. The triphasic curve all reached the nadir at 1 h, while the biphasic curve’s nadir time could be 1 h or 2 h. Regarding β-cell function, the curve with a nadir time of 2 h had significantly lower insulinogenic index and disposition index (Table 3). But there was no marked difference between the two groups for the HOMA-IR and Matsuda Index. Early glucose and insulin peak times were all associated with lower HOMA-IR (*p* < 0.001) and a higher disposition index (*p* < 0.001) (Tables 4 and 5).

**Table 3:** Demographic and metabolic characteristics of 1150 participants with OGTT glucose nadir at 60 min versus at 120 min

	OGTT glucose nadir at 60 min (*n* = 688)	OGTT glucose nadir at 120 min (*n* = 462)	P
Age (years)	35.5 ± 13.2	35.3 ± 13.8	0.824
Sex (male/female), n%	119 (17.3)/569 (82.7)	100 (21.6)/362 (78.4)	0.066
FBG (mmol/L)	5.2 ± 0.6	5.2 ± 0.6	0.069
30 min GLU(mmol/L)	8.2 ± 1.6	8.5 ± 1.6	0.004
2 h GLU(mmol/L)	7.1 ± 2.0	5.2 ± 1.6	<0.001
FINS	13.5 ± 7.4	13.4 ± 8.6	0.743
30 min INS	137.7 ± 67.5	102.3 ± 60.6	<0.001
2 h INS	92.6 ± 61.3	53.2 ± 42.3	<0.001
Glycemic status(%)			
NGT(%)	489 (71.1)	414 (89.6)	
IFG/IGT/IFG + IGT(%)	167 (24.3)	39 (8.4)	< 0.001
DM(%)	32 (4.7)	9 (1.9)	
Glucose AUC (mg·dL ^−1^· h ^−1^)	1175.3 ± 267.0	1190.0 ± 243.4	0.342
Insulin AUC(mg·dL ^− 1^· h ^− 1^)	14,349.1 ± 7572.7	13,402.2 ± 7358.6	0.036
HOMAIR	3.1 ± 1.8	3.1 ± 2.2	0.957
Matsuda Index	3.8 ± 2.5	3.8 ± 2.4	0.670
Insulinogenic index	49.7 ± 40.8	32.7 ± 29.7	<0.001
Disposition index	18.8 ± 19.0	13.1 ± 15.3	<0.001

Values are reported as the mean ± SD or n (%). *FBG* fasting blood glucose, *30 min GLU* glucose at 30 min, *2 h GLU* glucose at 120 min, *FINS* fasting insulin, *30 min INS* insulin at 30 min, *2 h INS* insulin at 1200 min, *NGT* normal glucose tolerance, *IFG* impaired fasting glucose, *IGT* impaired glucose tolerance, *DM* diabetes mellitus, *AUC* area under the curve

**Table 4:** Demographic and metabolic characteristics of 9059 participants with OGTT glucose peak at 30 min, 60 min versus 120 min

	OGTT glucose peak at 30 min (*n* = 4599)	OGTT glucose peak at 60 min (*n* = 3912)	OGTT glucose peak at 120 min (*n* = 548)	P
Age (years)	35.8 ± 12.8	40.1 ± 13.6	44.9 ± 14.9	<0.001
Sex (male/female), n%	974 (21.2)/3625 (78.8)	1178 (30.1)/2734 (69.9)	167 (30.5)/381 (69.5)	<0.001
FBG(mmol/L)	5.2 ± 0.6	5.7 ± 1.1	6.6 ± 2.0	<0.001
30 min GLU(mmol/L)	8.8 ± 1.6	9.8 ± 2.1	10.6 ± 3.0	<0.001
2 h GLU(mmol/L)	6.3 ± 1.5	8.9 ± 2.8	14.4 ± 4.3	<0.001
FINS	13.6 ± 7.7	15.6 ± 9.2	16.5 ± 10.3	<0.001
30 min INS	121.8 ± 63.6	82.0 ± 50.1	62.6 ± 48.3	<0.001
2 h INS	85.9 ± 58.8	121.5 ± 68.9	116.0 ± 73.2	<0.001
Glycemic status(%)				
NGT(%)	3749 (81.5)	1386 (35.4)	21 (3.8)	
IFG/IGT/IFG + IGT(%)	766 (16.7)	1693 (43.3)	109 (19.9)	< 0.001
DM(%)	84 (1.8)	833 (21.3)	418 (76.3)	
Glucose AUC (mg·dL ^−1^· h ^−1^)	1202.6 ± 212.3	1609.3 ± 385.4	2163.9 ± 643.5	<0.001
Insulin AUC(mg·dL ^−1^· h ^− 1^)	15,048.7 ± 7705.2	17,313.6 ± 8561.2	14,668.6 ± 8865.8	<0.001
HOMAIR	3.2 ± 1.9	4.1 ± 2.8	4.9 ± 3.9	<0.001
Matsuda Index	3.4 ± 2.1	2.6 ± 1.7	2.8 ± 2.1	<0.001
Insulinogenic index	36.1 ± 30.0	18.8 ± 16.2	15.0 ± 20.9	<0.001
Disposition index	15.6 ± 16.2	11.0 ± 12.4	8.6 ± 12.6	<0.001

Values are reported as the mean ± SD or n (%). *FBG* fasting blood glucose, *30 min GLU* glucose at 30 min, *2 h GLU* glucose at 120 min, *FINS* fasting insulin, *30 min INS* insulin at 30 min, 2 h *INS* insulin at 1200 min, *NGT* normal glucose tolerance, *IFG* impaired fasting glucose, *IGT* impaired glucose tolerance, *DM* diabetes mellitus, *AUC* area under the curve

**Table 5:** Demographic and metabolic characteristics of 9059 participants with OGTT insulin peak at 30 min, 60 min versus 120 min

	OGTT insulin peak at 30 min (*n* = 2935)	OGTT insulin peak at 60 min (*n* = 3729)	OGTT insulin peak at 120 min (*n* = 2279)	OGTT insulin peak at 180 min (*n* = 116)	P
Age (years)	36.1 ± 13.5	37.9 ± 13.1	41.3 ± 13.9	39.7 ± 14.4	<0.001
Sex (male/female), n%	610 (20.8)/2325 (79.2)	1073 (28.8)/2656 (71.2)	611 (26.8)/1668 (73.2)	25 (21.2)/91 (78.8)	<0.001
FBG(mmol/L)	5.2 ± 1.0	5.5 ± 1.0	5.9 ± 1.2	6.3 ± 1.6	<0.001
30 min GLU(mmol/L)	8.6 ± 1.7	9.4 ± 2.0	10.1 ± 2.0	10.2 ± 2.6	<0.001
2 h GLU(mmol/L)	6.9 ± 2.4	7.1 ± 2.6	10.3 ± 3.3	12.9 ± 4.2	<0.001
FINS	13.7 ± 7.9	14.8 ± 8.8	15.5 ± 8.8	17.2 ± 11.2	<0.001
30 min INS	133.0 ± 66.1	95.4 ± 54.9	71.3 ± 43.7	56.4 ± 35.1	<0.001
2 h INS	85.5 ± 59.7	89.6 ± 56.4	147.2 ± 70.6	117.0 ± 62.9	<0.001
Glycemic status(%)					
NGT(%)	2211 (75.3)	2514 (67.4)	424 (18.6)	7 (5.3)	
IFG/IGT/IFG + IGT(%)	549 (18.7)	893 (24.0)	1089 (47.8)	38 (32.7)	< 0.001
DM(%)	175 (6.0)	322 (8.6)	766 (33.6)	71 (61.9)	
Glucose AUC (mg·dL ^−1^· h ^−1^)	1239.8 ± 329.6	1389.2 ± 362.0	1738.5 ± 440.9	2070.4 ± 580.7	<0.001
Insulin AUC(mg·dL ^−1^· h ^− 1^)	14,403.2 ± 7536.1	16,046.0 ± 8141.5	17,980.4 ± 8741.5	17,006.3 ± 8977.3	<0.001
HOMAIR	3.3 ± 2.1	3.7 ± 2.6	4.2 ± 2.8	5.1 ± 4.5	<0.001
Matsuda Index	3.5 ± 2.2	2.9 ± 1.9	2.6 ± 1.8	2.8 ± 2.6	<0.001
Insulinogenic index	41.8 ± 33.4	23.9 ± 19.4	15.1 ± 13.5	11.7 ± 10.2	<0.001
Disposition index	17.4 ± 17.6	12.7 ± 13.5	9.0 ± 10.7	6.7 ± 7.1	<0.001

Values are reported as the mean ± SD or n (%). *FBG* fasting blood glucose, *30 min GLU* glucose at 30 min, *2 h GLU* glucose at 120 min, *FINS* fasting insulin, *30 min INS* insulin at 30 min, *2 h INS* insulin at 1200 min, *NGT* normal glucose tolerance, *IFG* impaired fasting glucose, *IGT* impaired glucose tolerance, *DM* diabetes mellitus, *AUC* area under the curve

### Change in the OGTT glucose curve shape and risk of impaired glucose metabolism

There were 635 participants who underwent OGTT twice. Table 5 shows baseline physical and metabolic characteristics of all the participants with a stable glucose response curve shape compared to those with unstable shape. Of the participants, 80.3% exhibited no change in shape between the baseline and the second OGTT (Table 6). Individuals who maintained a monophasic glucose response curve had the lowest rate of NGT, and those who maintained a multiphasic glucose response curve had the highest rate of NGT at baseline. Individuals with a stable monophasic glucose response shape had significantly higher fasting and 2hPG. Persistence of the monophasic shape was, in general, associated with worse insulin sensitivity and reduced β-cell function. Individuals whose glucose response curve changed from multiphasic to monophasic tended to have a higher rate of deterioration in glucose metabolism (Table 7).

**Table 6:** Demographic and metabolic characteristics of 502 participants with stable versus unstable OGTT-glucose response curve

	Stable		Unstable		*P* value
	Monophasic	Multiphasic	Monophasic to Multiphasic	Multiphasic to Monophasic	
N	371	9	55	67	
Age (years)	35.5 ± 12.3	37.0 ± 11.3	32.9 ± 13.2	35.1 ± 14.6	0.535
Sex (male/female, %)	62 (16.7)/309 (83.3)	2 (22.2)/7 (77.8)	9 (16.4)/46 (83.6)	9 (13.4)/58 (86.6)	0.878
FBG(mmol/L)	5.6 ± 0.9	5.1 ± 0.6	5.2 ± 0.6	5.2 ± 0.5	< 0.001
30 min GLU(mmol/L)	9.2 ± 1.7	8.2 ± 1.4	8.5 ± 1.3	8.3 ± 1.4	< 0.001
2 h GLU(mmol/L)	8.1 ± 2.7	5.6 ± 1.2	6.5 ± 1.6	6.5 ± 1.7	< 0.001
FINS	17.7 ± 9.2	11.1 ± 5.3	15.4 ± 10.0	16.8 ± 9.7	0.075
30 min INS	111.2 ± 65.3	152.8 ± 69.7	131.2 ± 74.8	150.2 ± 67.7	< 0.001
2 h INS	128.8 ± 72.2	69.3 ± 46.7	101.7 ± 66.9	97.0 ± 65.7	< 0.001
Glycemic status(n,%)					
NGT	181 (48.8)	9 (100)	42 (76.4)	51 (76.1)	
IFG/IGT/IFG + IGT	145 (39.1)	0	12 (21.8)	16 (23.9)	< 0.001
DM	45 (12.1)	0	1 (1.8)	0	
Glucose AUC (mg·dL ^−1^· h ^−1^)	1461.3 ± 366.0	1100.2 ± 83.1	1230.5 ± 231.0	1184.8 ± 208.6	< 0.001
Insulin AUC (mg·dL ^−1^· h ^− 1^)	19,184.5 ± 8734.1	13,817.2 ± 5128.2	17,677.0 ± 90,944.7	18,637.6 ± 8730.2	0.068
HOMA-IR	4.4 ± 2.6	2.6 ± 1.2	3.6 ± 2.4	3.9 ± 2.3	0.012
Matsuda Index	2.4 ± 1.6	3.8 ± 2.3	3.1 ± 2.0	3.1 ± 2.0	< 0.001
Insulinogenic index	29.6 ± 28.3	52.8 ± 34.1	37.7 ± 23.7	55.2 ± 56.7	< 0.001
Disposition index	17.2 ± 17.3	16.3 ± 7.0	19.2 ± 18.2	24.3 ± 20.4	0.025

Values are reported as the mean ± SD or n (%). *FBG* fasting blood glucose, *30 min GLU* glucose at 30 min, *2 h GLU* glucose at 120 min, *FINS* fasting insulin, *30 min INS* insulin at 30 min, *2 h INS* insulin at 1200 min, *NGT* normal glucose tolerance, *IFG* impaired fasting glucose, *IGT* impaired glucose tolerance, *DM* diabetes mellitus, *AUC* area under the curve

**Table 7:** The relationship between the change of the phase number and the change of the glycemic status

Glycemic status		Improve	Unchanging	Aggravation	Total
Change of the phase-number	Decrease	7 (10.4%)	49 (73.1%)	11 (16.4%)	67
	Unchanging	65 (17.1%)	245 (64.5%)	70 (18.4%)	380
	Increase	11 (20.0%)	38 (69.1%)	6 (10.9%)	55
Total		83	332	87	502

Values are reported as N (n%)

## Discussion

The present investigation revealed the following findings regarding the shape of OGTT curve in a large Chinese population: 1) multiphasic OGTT response curves were not rare in Chinese people, accounting for more than 10% of the population; 2) monophasic curves were more common in older people and in those with worse glycemic status; 3) individuals with monophasic curves had poorer β-cell function than individuals with multiphasic curves, despite having similar glycemic status; 4) individuals who were in NGT with a monophasic shape showed significantly worse insulin sensitivity, as reflected by the HOMA-IR and Matsuda Index, compared to patients with a multiphasic curve; 5) β-cell function was better in patients whose glucose concentration started to decrease at 60 min compared to later among the multiphasic curve group; and 6) the number of phases of the same subject could change at different times, and the number of phases increased with the improvement of glucose status.

In studies of nondiabetic individuals, the morphology of the monophasic glucose response curve is the dominant phenotype, up to 57–84% in adults [12, 13, 17, 22, 23] and 35–69% in obese youth at high risk for T2D [6, 7, 9, 10, 24]. Our study showed that about 88.3% of individuals had monophasic response curves and 11.7% had multiphasic response curves. Combining our present research with previous studies in youth [6, 10] and adults [12], the multiphasic group tends to be associated with younger age compared with the monophasic group. Our study further found that there was little difference between the two glucose curve shape groups when the glycemic status reached diabetes, and both young and old individuals had an extremely low proportion of multiphasic OGTT response curve.

Cross-sectional studies in youths [6–13] and adults [11–13, 23] showed that the shape of the OGTT glucose response curve could indicate insulin sensitivity and β-cell function, as well as differentiate type 2 diabetes risk. Obese youths with monophasic glucose response curves were worse in both hepatic and peripheral insulin sensitivity measured by the clamp method compared with the biphasic group, as well as in β-cell function, which was indicated by impaired disposition index as a result of lacking in a compensatory increase in first and second-phase insulin secretion [6]. Evidence from patients with suspected gestational diabetes who underwent 3 h OGTT showed that a greater number of phases in the OGTT glucose response curve was associated with a healthier metabolic state, which suggests that a biphasic response curve may be associated with a lower incidence of prediabetes and T2D [13]. In our study, β-cell function was better in individuals with multiphasic glucose response curve. As for insulin sensitivity, the difference was still significant in the NGT group, but the difference did not remain significant in the diabetes group. The most likely reason is that defects in β-cell function are more severe in Chinese patients with diabetes than those in Europeans or Americans, resulting in more serious deficiencies in insulin secretion [25].

Am American study conducted in adult patients showed that the baseline and subsequent glucose concentrations in the OGTT could stratify the risk for progression to T2D; that is, a faster return to the FPG concentration may suggest a lower risk of T2D [22]. Our study further found that individuals with multiphasic curves whose plasma glucose concentration reached the lowest point at 60 min had better β-cell function than those with a nadir at 120 min. Consistent with previous studies [26], we found that the earlier the glucose/insulin peak in the OGTT curve, the better the β-cell function.

Our data showed that individuals whose response glucose curve changed from multiphasic to monophasic during follow-up were more prone to deteriorate in glycemic status than those whose glucose response curves changed from monophasic curves to multiphasic curves. This result is supported by several longitudinal studies. A 7–8 year longitudinal study demonstrated that pre-diabetic patients with monophasic curves had twice the incidence of diabetes as those with biphasic curves, despite similar fasting and 2hPG concentrations [22]. Individuals with a monophasic curve at baseline and those whose patterns changed from biphasic to monophasic had an increased risk for impaired glucose metabolism [14]. We also found that individuals with a persistent monophasic curve had worse insulin sensitivity and β-cell function than those with other forms at baseline.

The strengths of the present investigation include the following: 1) it is the first large-scale (up to 10,000) investigation of the relationship between the OGTT glucose response curve and insulin resistance/β-cell function in Chinese people; 2) the study included people with different glucose metabolic states and across different age groups. Potential perceived limitations would be that we have no anthropometric data, such as body mass index (BMI) and waist circumference, which could have an influence on glycemic status. But previous research showed that the OGTT response curve shape remained strongly associated with insulin sensitivity and β-cell function after adjusting for BMI, blood pressure, and waist circumference [6, 8]. In addition, our research follows the standard methods used in China. The OGTT glucose response curve shape was determined by data only at 0, 30, 60, 120, and 180 min, lacking 90 min glucose data, which may have led to an underestimation in the phase of the curve. Investigations of the change in patterns or shapes of the OGTT glucose response curves did not have regular follow-up.

## Conclusions

In summary, the present study is the first to demonstrate that in a large Chinese population, the monophasic OGTT glucose response curve was associated with reduced β-cell function, higher HOMA-IR, and older age. However, prospective longitudinal studies are needed to verify the usefulness of the OGTT glucose response curve in predicting progression to prediabetes or T2D in Chinese. Further, it remains essential to examine whether any factors could shift the OGTT glucose response curve from monophasic to multiphasic.

## Data Availability

The datasets used and analyzed during the current study are available from the corresponding author on reasonable request.

## Abbreviations

2hPG: 2-h plasma glucose
BMI: Body mass index
DM: Diabetes mellitus
FINS: Fasting serum insulin
FPG: Fasting plasma glucose
IFG: Impaired fasting glucose
IGT: Impaired glucose tolerance
NGT: Normal glucose tolerance
OGTT: Oral glucose tolerance test
T2D: Type 2 diabetes

## References

[1] Rehman K, Akash MS (2016). Mechanisms of inflammatory responses and development of insulin resistance: how are they interlinked?. J Biomed Sci.

[2] Rehman K, Akash MSH (2017). Mechanism of generation of oxidative stress and pathophysiology of type 2 diabetes mellitus: how are they interlinked?. J Cell Biochem.

[3] Schwartz SS, Epstein S, Corkey BE, Grant SF, Gavin JR, Aguilar RB (2016). The time is right for a new classification system for diabetes: rationale and implications of the beta-cell-centric classification schema. Diabetes Care.

[4] Heise T, Zijlstra E, Nosek L, Heckermann S, Plum-Morschel L, Forst T (2016). Euglycaemic glucose clamp: what it can and cannot do, and how to do it. Diabetes Obes Metab.

[5] Jia W, Weng J, Zhu D, Ji L, Lu J, Zhou Z (2019). Standards of medical care for type 2 diabetes in China 2019. Diabetes Metab Res Rev.

[6] Kim JY, Michaliszyn SF, Nasr A, Lee S, Tfayli H, Hannon T (2016). The shape of the glucose response curve during an oral glucose tolerance test heralds biomarkers of type 2 diabetes risk in obese youth. Diabetes Care.

[7] Bervoets L, Mewis A, Massa G (2015). The shape of the plasma glucose curve during an oral glucose tolerance test as an indicator of Beta cell function and insulin sensitivity in end-pubertal obese girls. Horm Metab Res.

[8] Herrera-Martinez AD, Enes P, Martin-Frias M, Roldan B, Yelmo R, Barrio R (2017). The monophasic pattern in oral glucose tolerance test as a predictive risk factor of type 2 diabetes in obese paediatric patients. An Pediatr (Barc).

[9] Kim JY, Coletta DK, Mandarino LJ, Shaibi GQ (2012). Glucose response curve and type 2 diabetes risk in Latino adolescents. Diabetes Care.

[10] Nolfe G, Spreghini MR, Sforza RW, Morino G, Manco M (2012). Beyond the morphology of the glucose curve following an oral glucose tolerance test in obese youth. Eur J Endocrinol.

[11] Kanauchi M, Kimura K, Kanauchi K, Saito Y (2005). Beta-cell function and insulin sensitivity contribute to the shape of plasma glucose curve during an oral glucose tolerance test in non-diabetic individuals. Int J Clin Pract.

[12] Tschritter O, Fritsche A, Shirkavand F, Machicao F, Haring H, Stumvoll M (2003). Assessing the shape of the glucose curve during an oral glucose tolerance test. Diabetes Care.

[13] Tura A, Morbiducci U, Sbrignadello S, Winhofer Y, Pacini G, Kautzky-Willer A (2011). Shape of glucose, insulin, C-peptide curves during a 3-h oral glucose tolerance test: any relationship with the degree of glucose tolerance?. Am J Physiol Regul Integr Comp Physiol.

[14] Manco M, Nolfe G, Pataky Z, Monti L, Porcellati F, Gabriel R (2017). Shape of the OGTT glucose curve and risk of impaired glucose metabolism in the EGIR-RISC cohort. Metabolism..

[15] Alberti KG, Zimmet PZ (1998). Definition, diagnosis and classification of diabetes mellitus and its complications. Part 1: diagnosis and classification of diabetes mellitus provisional report of a WHO consultation. Diabet Med.

[16] WHO/IDF consultation. Definition and diagnosis of diabetes mellitus and intermediate hyperglycemia.2006. https://www.who.int/diabetes/publications/Definition%20and%20diagnosis%20of%20diabetes_new.pdf

[17] Ismail HM, Xu P, Libman IM, Becker DJ, Marks JB, Skyler JS (2018). Type 1 diabetes TrialNet study G. the shape of the glucose concentration curve during an oral glucose tolerance test predicts risk for type 1 diabetes. Diabetologia..

[18] Matthews DR, Hosker JP, Rudenski AS, Naylor BA, Treacher DF, Turner RC (1985). Homeostasis model assessment: insulin resistance and beta-cell function from fasting plasma glucose and insulin concentrations in man. Diabetologia..

[19] Matsuda M, DeFronzo RA (1999). Insulin sensitivity indices obtained from oral glucose tolerance testing: comparison with the euglycemic insulin clamp. Diabetes Care.

[20] Kosaka K, Hagura R, Kuzuya T, Kuzuya N (1974). Insulin secretory response of diabetics during the period of improvement of glucose tolerance to normal range. Diabetologia..

[21] Retnakaran R, Qi Y, Goran MI, Hamilton JK (2009). Evaluation of proposed oral disposition index measures in relation to the actual disposition index. Diabet Med.

[22] Abdul-Ghani MA, Lyssenko V, Tuomi T, Defronzo RA, Groop L (2010). The shape of plasma glucose concentration curve during OGTT predicts future risk of type 2 diabetes. Diabetes Metab Res Rev.

[23] Trujillo-Arriaga HM, Roman-Ramos R (2008). Fitting and evaluating the glucose curve during a quasi continuous sampled oral glucose tolerance test. Comput Biol Med.

[24] Arslanian S, El Ghormli L, Young Kim J, Bacha F, Chan C, Ismail HM (2019). The shape of the glucose response curve during an oral glucose tolerance test: forerunner of heightened glycemic failure rates and accelerated decline in beta-cell function in TODAY. Diabetes Care.

[25] Jia W (2017). Diabetes research in China: making progress. Lancet Diabetes Endocrinol.

[26] Cree-Green M, Xie D, Rahat H, Garcia-Reyes Y, Bergman BC, Scherzinger A (2018). Oral glucose tolerance test glucose peak time is most predictive of prediabetes and hepatic steatosis in obese girls. J Endocr Soc.

